# Supplementary material for: The ESCRT-0 protein HRS regulates hepatocellular lipid droplet catabolism
Source: bioRxiv. 2025 Nov 13:2025.11.13.686840. Preprint. [Version 1] doi: 10.1101/2025.11.13.686840 (PMC12642657; doi:10.1101/2025.11.13.686840)
Supplement: Supplement 3 — Figure S3. A. Confocal micrographs representing endogenous HRS by immunofluorescence (green) in AML12 cells where LDs are stained with ORO (red). B. Confocal micrographs representing AML12 cells transfected with an HRS-overexpression plasmid (red) where LDs are stained with MDH. C. Graph depicting organelle localization of HRS protein from mouse livers along a sucrose gradient.21 [file media-3.pdf]

A.

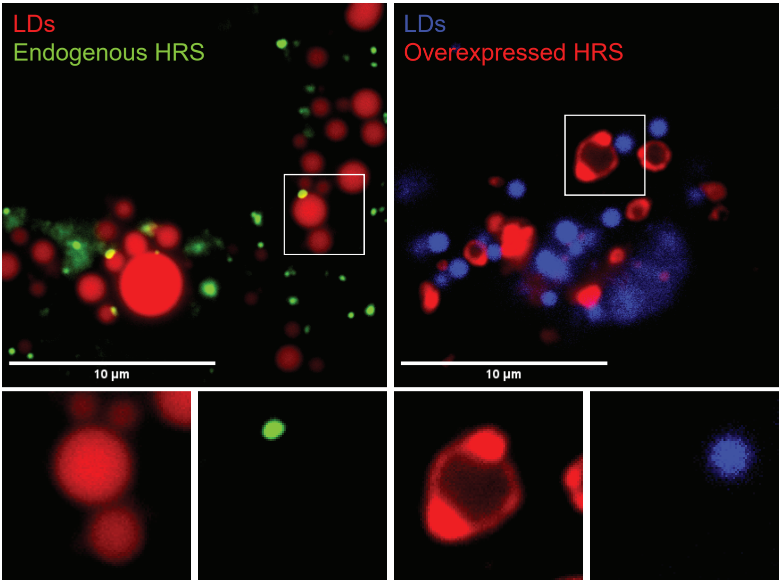

B.

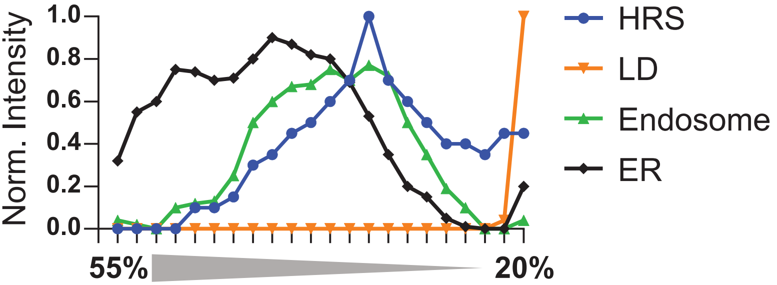

Figure S3.

A. Confocal micrographs representing endogenous HRS by immunofluorescence (green) in AML12 cells where LDs are stained with ORO (red). B. Confocal micrographs representing AML12 cells transfected with an HRS-overexpression plasmid (red) where LDs are stained with MDH. C. Graph depicting organelle localization of HRS protein from mouse livers along a sucrose gradient.[21]
